# The correlation between erectile dysfunction and metabolic syndrome in an Indian population: A cross-sectional observational study

**DOI:** 10.1080/2090598X.2019.1600990

**Published:** 2019-04-24

**Authors:** Rajeev Sood, Dushiant Sharma, Hemant Goel, Nikhil Khattar, Bindu Kulshreshtha, Kunal K. Singh

**Affiliations:** a Department of Urology and Renal Transplant, Pgimer and Dr RML Hospital, New Delhi, India; b Department of Endocrinology, Pgimer and Dr RML Hospital, New Delhi, India

**Keywords:** Erectile dysfunction, Metabolic syndrome, Insulin resistance, IIEF-5, Indian population

## Abstract

**Objective**: To evaluate the relationship between erectile dysfunction (ED), based on the five-item International Index of Erectile Function questionnaire (IIEF-5), and presence of metabolic syndrome (MetS) or its components based on Adult Treatment Panel III guidelines. We also explored the impact of increasing insulin resistance (IR), as calculated using the Homeostatic Model Assessment for Insulin Resistance (HOMA-IR) equation, on severity of ED. Pathophysiological links between ED and testosterone were re-evaluated.

**Patients and methods**: In all, 357 patients with ED were evaluated; 53 patients with primary, psychogenic, surgical, post-traumatic or drug-induced ED were excluded. The remaining 304 patients were evaluated after obtaining written informed consent. The Institutional Review Board approved the study. We assessed comorbidities, IIEF-5 scores, lower urinary tract symptoms (LUTS) based on International Prostate Symptom Score (IPSS), blood sugars, lipid and hormonal profiles, and vitamin D3 levels. Further evaluation was done when indicated.

**Results**: In all, 171 patients had MetS and 134 had pre-existing comorbidities (diabetes mellitus, 58; hypertension, 73; coronary artery disease, 13). The mean (SD) age was 44.6 (9.21) years and IIEF-5 score was 13.81 (3.17). ED severity was significantly correlated with presence of MetS. On multivariate analysis, there were significant correlations between ED and waist circumference, serum triglycerides, and fasting blood sugar. There was a statistically significant positive correlation between serum testosterone and IIEF-5 score (*r* = +0.292). The mean (SD) IR value (using the HOMA-IR formula) was 2.64 (2.87), which was statistically and negatively correlated with IIEF-5 scores (*r* = – 0.398). Receiver operating characteristic analysis showed that an IIEF-5 score of <14 predicted MetS and a HOMA-IR value of >2.1778 predicted MetS.

**Conclusion**: MetS or its components were present in 56.25% of the patients. Therefore presence of ED merits further evaluation for presence of MetS. This may help to prevent catastrophic and life-threatening consequences of MetS.

**Abbreviations:** BMI: body mass index; CRP: C-reactive protein; CVD: cardiovascular disease; DBP: diastolic blood pressure; DM: diabetes mellitus; ED: erectile dysfunction; FBS: fasting blood sugar; HDL: high-density lipoprotein; HOMA-IR- Homeostatic Model Assessment for Insulin Resistance; HTN: hypertension; IIEF-5: five-item version of the International Index of Erectile Function; IR: insulin resistance; LDL: low-density lipoprotein; LUTS: lower Urinary Tract Symptoms; MetS: metabolic syndrome; NO: nitric oxide; OR: odds ratio; PPBS: post-prandial blood sugar; ROC: receiver operating characteristic; SBP: systolic blood pressure; TG: triglyceride; WC: waist circumference

## Introduction

Erectile dysfunction (ED) has become one of the most common chronic disorders affecting 100 million men worldwide [1] and has attained even more significance as it has been labelled a sentinel marker for cardiovascular disease (CVD) and metabolic disorders. Metabolic syndrome (MetS) is a cluster of disorders leading to the development of CVD and diabetes mellitus (DM) [2]. It consists of central obesity, hypertension (HTN), dyslipidaemia, and impaired glucose tolerance. There are many proposed mechanisms for explaining the correlation between ED and MetS.

### Endothelial dysfunction

ED is considered a vascular disease in many patients with underlying endothelial dysfunction [3], with the involvement of the nitric oxide (NO) pathway forming the common link between ED, CVD and MetS. Endothelial dysfunction in ED usually affects smaller vessels; therefore, ED could act a sensitive predictor for eventual development of large vessel endothelial dysfunction [4].

### Chronic inflammation

Low-grade chronic subclinical inflammation is another factor that affects endothelial function and has been known to be associated with both ED and MetS. Chronic inflammation leads to increased expression of pro-inflammatory cytokines. Plasma levels of C-reactive protein (CRP) are significantly higher in patients with ED compared with subjects without ED matched for age and coronary risk score [5]. Increases in pro-inflammatory cytokines have also been reported time and again in obese and diabetic patients who have underlying metabolic dysfunction in majority of the cases [6,7].

### Hyperglycaemia

Hyperglycaemia, which is one of the components of MetS, induces a series of cellular events that increase the production of reactive oxygen species resulting in inactivation of NO [8] and glycation of penile cavernosal tissue that leads to impairment of collagen turnover and later to ED [9].

### Hypoandrogenism

Low androgen levels are associated with ED, MetS, DM, and CVD [10,11]. Hypogonadism caused by the MetS can cause ED through altered testosterone:oestrogen levels, which leads to hypogonadotrophic hypogonadism and a decreasing testosterone level that impairs NO synthesis.

There is an enormous amount of literature in the Western world regarding the incidence of ED and its effect on cardiovascular and metabolic health of a patient. But India, despite having the one of the highest numbers of patients with DM and MetS in the world and despite the growing incidence of obesity and CVD, does not have many studies on the role of ED as a precursor and a marker of MetS. The presence of ED would serve as a cheap and easy-to-recognise marker for detection of occult and yet to develop MetS, DM and CVD in our overburdened healthcare system.

Studies that have focused on the association between ED and MetS in the Indian population are sparse. Therefore, the present study aimed to intensively evaluate the relationship of ED and MetS in Indian men.

## Patients and methods

In all, 357 patients presenting to the Outpatient Department with the complaint of ED were enrolled for the study. The Institutional Review Board of our hospital approved the study. All of the men gave informed consent to participate in the study. All patients completed the five-item version of the International Index of Erectile Function (IIEF-5) questionnaire to detect ED. The five parameters of sexual function evaluated included: erectile function, orgasmic function, sexual desire, intercourse satisfaction, and overall satisfaction. Each item was rated on a 5-point scale. Men with a score of *≤*21 (range 5–25) were considered to have ED. The IIEF-5 score was divided into five groups: 1–7, severe ED; 8–11, moderate ED; 12–16, mild–moderate ED; 17–21, mild ED; and 22–25, no ED [12]. For our present study, patients were sub-grouped into two categories: mild/mild–moderate ED (IIEF-5 score 12–21) and moderate/severe ED (IIEF-5 score 5–11). Patients with psychogenic ED, post-surgical or post-traumatic ED, primary ED, chronic kidney disease or drug-induced ED were excluded. In all, 53 patients were excluded [post-surgical (20 patients), history of pelvic fracture (six), chronic kidney disease (eight), use of anti-depressants (17), prolactinoma (one), primary ED (one)]. Thus, 304 patients were finally evaluated after the exclusion criteria were applied.

Complete personal and medical histories were taken for each patient. Factors such as age, level of physical activity, smoking habits, past history of CVD, HTN, DM, thyroid disorder, and medications were assessed by direct questioning. LUTS, if any, were graded according to the IPSS. Body weight was measured by an electronic weighing machine and height was measured using a stadiometer with the patient barefoot and fully erect, with the head in the Frankfurt plane; the back of the head, thoracic spine, buttocks, and both heels together touching the vertical axis of the stadiometer. Body mass index (BMI) was then calculated using the formula weight (in kilograms)/height^2^ (in metres). The waist circumference (WC) was measured midway between the lowest rib and the iliac crest. Blood pressure was measured using a mercury sphygmomanometer placed on the right arm of the patients, with the patients in a comfortable sitting position after 5 min of rest, on two different occasions, at least a week apart. The following investigations were conducted: fasting blood sugar (FBS), post-prandial blood sugar (PPBS), glycated haemoglobin (HbA1c; if FBS or PPBS were abnormal), fasting plasma insulin levels, liver and renal function tests, lipid profile, thyroid profile, total and free testosterone levels (if required), FSH, LH, prolactin, CRP, electrocardiogram, and imaging studies (e.g. penile Doppler), were performed whenever deemed necessary. MetS was diagnosed based on Adult treatment Panel III criteria [13], which include the presence of at least three of the following characteristics for diagnosis of MetS: abdominal obesity (WC >102 cm in men and >88 cm in women), elevated triglycerides (TGs >150 mg/dL), reduced levels of high-density lipoprotein (HDL) cholesterol (HDL-cholesterol <40 mg/dL in men and <50 mg/dL in women), high blood pressure (blood pressure >130/85 mmHg), and high FBS (>110 mg/dL). Insulin resistance (IR) was calculated using the Homeostatic Model Assessment for Insulin Resistance (HOMA-IR) equation: [fasting plasma insulin (µIU/mL) × FBS (mg/dL)]/405.

### Statistical analysis

Quantitative variables were compared using the unpaired *t*-test/Mann–Whitney test and qualitative variables were correlated using the chi-squared test/Fisher’s exact test. The Pearson correlation coefficient/Spearman rank correlation coefficient was used to assess the association of IIEF-5 with CRP and testosterone and the correlation between HOMA-IR and IIEF-5. A receiver operating characteristic (ROC) curve was used to calculate the IIEF-5 score threshold for MetS and its components, and threshold value of HOMA-IR for MetS. The area under the ROC curve predicted the accuracy of the IIEF-5 score for presence of MetS or its abnormal components. Accuracy was subdivided into the following groups: 0.90–1, excellent; 0.80–0.90, good; 0.70–0.80, fair; 0.60–0.70, poor; 0.50–0.60, fail. A *P *< 0.05 was considered statistically significant.

## Results

**Figure 1.**
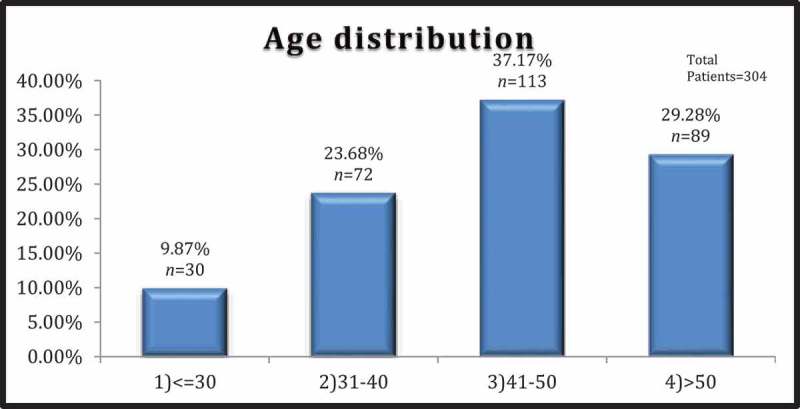
Age distribution of the study population.

**Figure 2.**
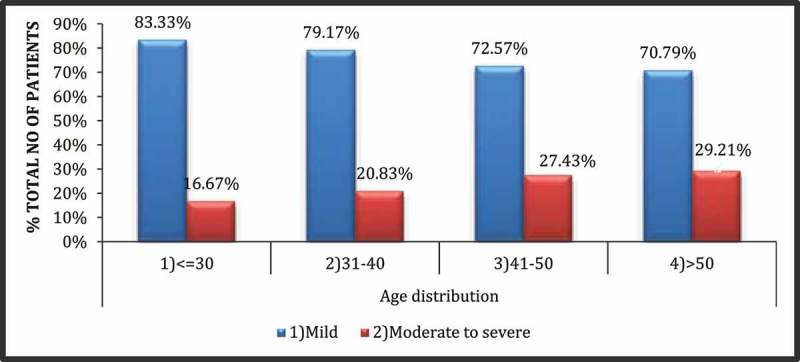
Correlation between age groups and severity of ED (mild ED IIEF-5 score = 12–21, moderate/severe ED IIEF-5 score = 5–11).

Table 1 shows the anthropometric and biochemical variables of 304 patients. All the parameters, age, IIEF-5 score, IPSS, BMI, diastolic blood pressure (DBP), systolic blood pressure (SBP), WC, FBS, fasting insulin, IR (HOMA‑IR), TGs, low-density lipoprotein (LDL), HDL, cholesterol, testosterone, and CRP, were highly variable in all patients. The mean (SD) age was 44.6 (9.21) years, with the majority in the 41–50 year age group (113 of 304). The breakdown of patients according to different age groups is shown in Figure 1. The severity of ED increased with increasing age (Figure 2) but this relationship was not statistically significant (*P* = 0.402). The mean (SD) WC, FBS, SBP, DBP, HDL, and TG were 100.11 (3.03) cm, 119.02 (44.66) mg/dL, 135.22 (16.82) mmHg, 81.47 (7.8) mmHg, 38.41 (6.87) mg/dL, and 150.88 (47.09) mg/dL, respectively. The mean (SD) IIEF‑5 score was 13.81 (3.17); 227 patients (74.67%) had mild/mild–moderate ED (IIEF-5 score 12–21) and 77 patients (25.32%) patients had moderate–severe ED (IIEF-5 score 5–11).

**Table 1.** General characteristic of the study population.

Variable (normal range)	Sample size, *n*	Mean (SD; range)	Median (interquartile range)
Anthropometric data			
Age, years	304	44.6 (9.21; 25–60)	45 (37.5–52)
IIEF-5 score	304	13.81 (3.17; 5–20)	14 (11–17)
IPSS (only in patients with LUTS)	199	11.16 (4.54; 2–29)	11 (8–14)
BMI, kg/m^2^	304	25.64 (2.36; 19–34)	25.6 (24.4–27.2)
SBP, mmHg	304	135.22 (16.82; 100–188)	134 (120–145)
DBP, mmHg	304	81.47 (7.8; 56–98)	82 (76–88)
WC, cm	304	100.11 (3.03; 86–112)	100 (99–102)
Biochemical parameters			
TGs (50–150 mg/dL)	304	150.88 (47.09; 53–398)	159.5 (99–187)
FBS (70–110 mg/dL)	304	119.02 (44.66; 84–532)	114 (100–126)
PPBS (90–160 mg/dL)	304	164.02 (65.39; 92–623)	154 (134–178)_
HDL (40–65 mg/dL)	304	38.41 (6.87; 23–55)	37.5 (33–44)
LDL (50–150 mg/dL)	304	100.77 (30.51; 29–189)	91 (79–123)
Total cholesterol(130–230 mg/dL)	304	175.41 (30.28; 94–275)	176 (156–201)
Testosterone (2.49–28.2 nmol/L)	304	12.86 (4.05; 3.45–26.5)	12.3 (10.3–15.3)
FSH (1.55–9.74 µIU/mL)	304	7.53 (2.1; 1.17–10.1)	8.3 (6.8–9.1)
Prolactin (3–18.6 ng/mL)	304	7.86 (2.21; 4–17.6)	7.7 (6.7–8.6)
Vitamin D_3_ (30–56 ng/mL)	304	13.35 (7.52; 5.64–54.5)	11.4 (9.2–13.8)
CRP (0–1.0 mg/dL)	304	0.65 (2.59; 0.01–26.1)	0.14 (0.09–0.70)
Urea (15–45 mg/dL)	304	22.66 (5.51; 14–38)	21 (19–24.5)
Creatinine (0.6–1.2 mg/dL)	304	0.8 (0.22; 0.3–1.2)	0.75 (0.7–1.0)

In all, 134 patients (44.08%) had pre-existing comorbidities such as DM, HTN, chronic obstructive pulmonary disease, CVD. HTN was the most common pre-existing comorbidity (73 patients). The data about smoking was not included in the final analysis as the duration of smoking, number of cigarettes smoked per day, and other data related to smoking could not be compiled because of poor recall of the patients, poor educational status and variation in type of tobacco exposure whether cigarette smoke, beedi smoke, use of hookah, or presence of second-hand exposure to tobacco.

**Figure 3.**
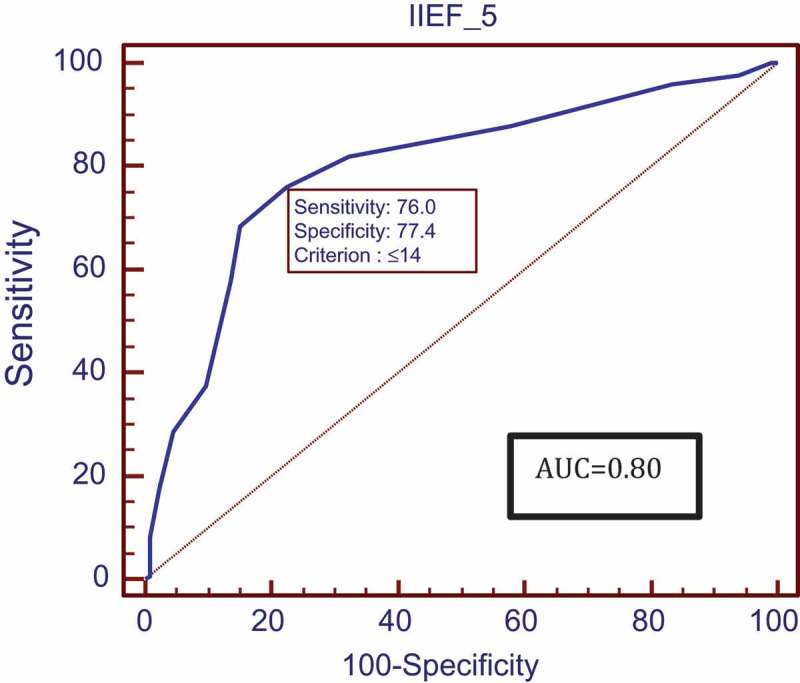
ROC analysis of IIEF-5 score thresholds predicting MetS.

**Figure 4.**
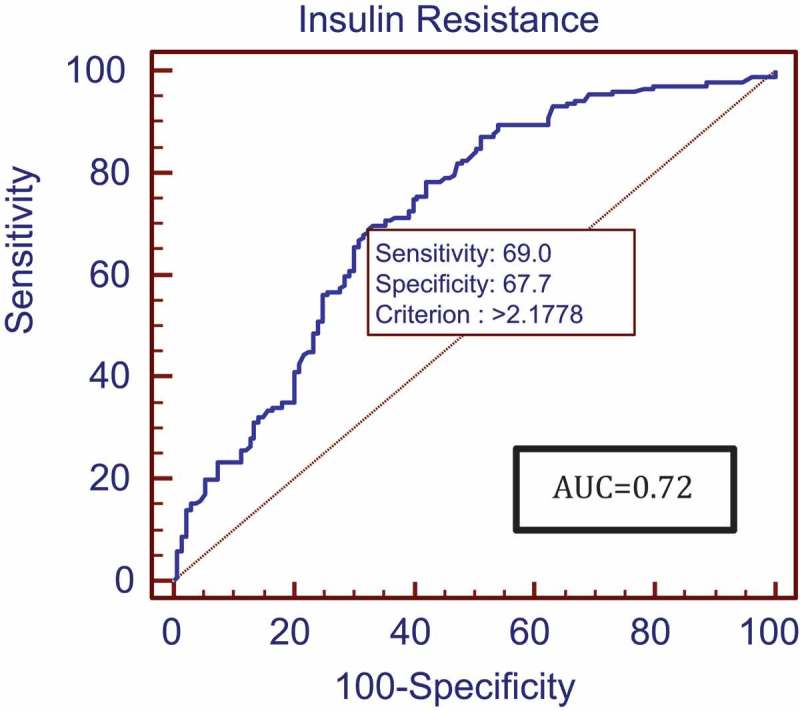
ROC analysis for HOMA-IR value thresholds predicting MetS.

**Figure 5.**
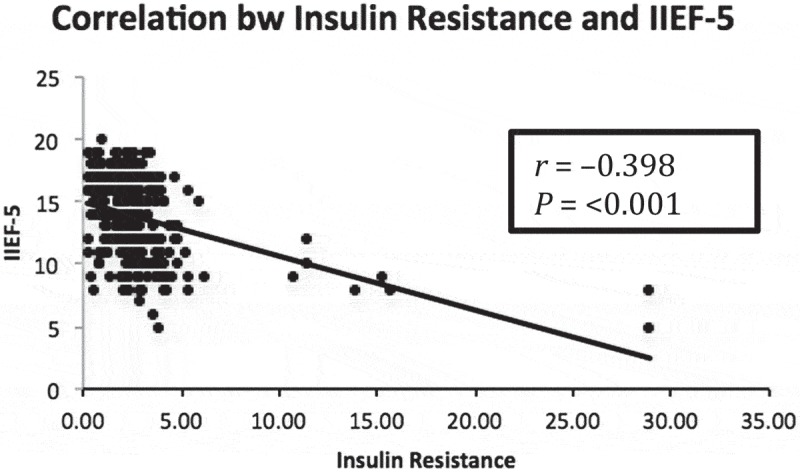
Scatter diagram of the correlation between ED (IIEF-5 score) and IR (HOMA-IR value) (*r* = correlation coefficient).

**Figure 6.**
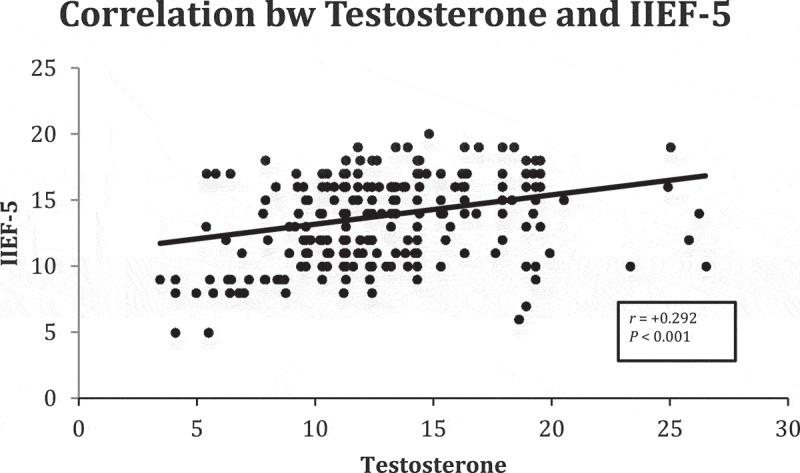
Scatter diagram of the correlation between IIEF-5 score and mean testosterone level (*r* = correlation coefficient).

The mean (SD) IPSS was 11.16 (4.54) calculated in 199 patients with LUTS. Patients with higher urinary distress with higher IPSSs had higher degrees of ED with low IIEF-5 scores. However, the relationship between IIEF-5 scores and IPSSs was not statistically significant (*P* = 0.116). MetS was diagnosed in 171 (56.25%) patients. The mean (SD) IIEF-5 score for patients with and without MetS was 12.39 (2.94) and 15.62 (2.47), respectively, showing lower IIEF-5 scores in patients with MetS. This correlation was found to be significant (*P* < 0.001). The ROC analysis revealed that an IIEF-5 score of ≤14 predicted the presence of MetS (area under the curve 0.80, good accuracy) (Figure 3). All components of MetS were correlated with severity of ED but statistical significance was not reached for the correlation between ED and HDL. After performing univariate logistic regression (Table 2), abnormal BP, WC, TGs and FBS increased the risk of severe ED with odds ratios (ORs) of 1.921, 2.731, 3.147 and 5.62, respectively; but on multivariate analysis (Table 3) only WC, TGs and FBS significantly increased the risk of severe ED with ORs of 2.069, 3.355 and 5.732, respectively. A HOMA-IR value threshold of >2.1778 predicted the presence of MetS with a sensitivity of 69% and specificity of 67.7% based on ROC analysis (Figure 4). The HOMA-IR value was strongly and negatively correlated to the severity of ED based on IIEF-5 scores [correlation coefficient (*r*) = – 0.398; *P* < 0.001], meaning that higher HOMA-IR values with greater IR were associated with lower IIEF-5 scores with more severe ED (Figure 5). The mean (SD) serum testosterone level was 12.86 (4.05) nmol/L and was significantly associated with severity of ED. Patients with more severe ED had lower serum testosterone values (*P* < 0.001) and a positive correlation coefficient with IIEF-5 scores (*r* = +0.292; Figure 6). Although patients with MetS had lower serum testosterone levels, the relationship between serum testosterone and MetS did not reach statistical significance (*P* = 0.251)

**Table 2.** Univariate analysis.

Variable (normal range)	β coefficient	Standard error	*P*	OR (95% CI)
BP (mmHg)	0.653	0.308	0.034	1.921 (1.051–3.512)
WC (cm)	1.005	0.321	0.002	2.731 (1.455–5.125)
TGs (50–150 mg/dL)	1.146	0.305	<0.001	3.147 (1.730–5.724)
FBS (70–110 mg/dL)	1.726	0.332	<0.001	5.620 (2.931–10.773)
HDL (40–65 mg/dL)	0.478	0.287	0.096	1.612 (0.919–2.827)

**Table 3.** Multivariate analysis.

Variable (normal range)	β coefficient	Standard error	*P*	OR (95% CI)
WC (cm)	0.727	0.358	0.042	2.069 (1.026–4.172)
TGs (50–150 mg/dL)	1.210	0.326	<0.001	3.355 (1.769–6.361)
FBS (70–110 mg/dL)	1.746	0.345	<0.001	5.732 (2.918–11.261)

## Discussion

In recent years, ED has been recognised as a sentinel marker for the detection of HTN, DM, dyslipidaemia, and CVD in their occult or subclinical forms. Multiple Western studies have shown some correlation between MetS and ED. For example, Seftel et al. [14] concluded that ED shared common risk factors with CVD. Esposito et al. [15] reported that men with MetS had an increased prevalence of ED. But studies in the Indian subcontinent are still sparse.

In our present study, MetS was diagnosed in 171 (56.25%) patients. The mean (SD) IIEF-5 score for patients with and without MetS was 12.39 (2.94) and 15.62 (2.47), respectively, showing significantly lower IIEF-5 scores in patients with MetS (*P* < 0.001). A similar finding was seen in a Taiwanese study by Yeh et al. [16], where the mean (SD) IIEF-5 score for patients with and without MetS was 12.7 (5.7) and 15.8 (7.0), respectively (*P* = 0.022).

Even though it appears that ED and MetS are closely correlated, it is not clear which components of MetS are actually responsible for worsening erectile function. In our present study, after multivariate analysis the factors significantly affecting erectile function were FBS, TG, and WC. However, Sanjay et al. [17] found that HDL, TGs and fasting plasma glucose significantly affected erectile function, with HDL conferring the strongest effect on erectile function.

IR has been found to be the central tenet of MetS (also known as IR syndrome). We calculated IR using the HOMA-IR equation with values >2.1778 predicting the presence of MetS. Different values have been suggested for different populations. In a study by Russo et al. [18], IR was found to be a significant risk factor for the development of ED. Singh et al. [19] conducted a study of 691 healthy adolescents in which a HOMA-IR value threshold of 2.5 provided the maximum sensitivity and specificity in diagnosing MetS, whereas Yoo et al. [20] calculated a HOMA IR value threshold of 2.63 for diagnosing MetS. Therefore, various HOMA-IR thresholds have been suggested by different studies; however, all patients with suspected organic ED can have their HOMA-IR values calculated and HOMA-IR value thresholds >2 may be used as marker for further evaluation for the presence of MetS.

Serum testosterone values were non-significantly low in patients with MetS in our present study (*P* = 0.251). This non-significant correlation could be explained by the lower average age of patients in our present study [mean (SD) 44.6 (9.21) years] compared to other studies, where the average age of the patient population was >50 years with increasing age-related deficiency of androgens. Testosterone levels correlated positively and significantly with IIEF-5 scores (*r* = 0.292; *P* < 0.001). Corona et al. [21] evaluated the impact of serum testosterone on the severity of ED and MetS. They found that low testosterone was associated with higher WC and TG levels, and an increased prevalence of MetS, along with an inverse relationship between testosterone levels and the prevalence of severe ED. Amidu et al. [22] also suggested that low testosterone was significantly associated with presence of sexual dysfunction (*P* = 0.025). All of these studies, combined with our present study, point to the important role of testosterone in the maintenance of the metabolic milieu and the role of hypoandrogenism in the development of manifestations of MetS.

Our present study has thus indicated that ED and MetS are closely inter-related, with the presence of ED predicting the presence of MetS. Both these diseases affect the quality of life of a large number of Indian males.

## Conclusion

It has thus become increasingly important to conduct a thorough evaluation of all patients with suspected organic ED. This could help in diagnosing MetS early enough so that all of its life threatening sequelae such as DM, HTN, and CVD, could be prevented in their nascent stage. This management strategy for ED has immense potential in a developing country like ours, especially in the area of preventive medicine and men’s health. Further studies should include analyses of both evaluations of patients along with treatment and final outcome of such patients, so that each centre can provide full specialised care from diagnosis to final treatment of these patients. This would usher in the era of men’s health as a separate and specific subspecialty
